# Epidemic spreading in time-varying community networks

**DOI:** 10.1063/1.4876436

**Published:** 2014-05-14

**Authors:** Guangming Ren, Xingyuan Wang

**Affiliations:** 1 School of Electronic & Information, Guangdong Polytechnic Normal University, Guangzhou 510665, China; 2 Faculty of Electronic Information & Electrical Engineering, Dalian University of Technology, Dalian 116024, China

## Abstract

The spreading processes of many infectious diseases have comparable time scale as the network evolution. Here, we present a simple networks model with time-varying community structure, and investigate susceptible-infected-susceptible epidemic spreading processes in this model. By both theoretic analysis and numerical simulations, we show that the efficiency of epidemic spreading in this model depends intensively on the mobility rate *q* of the individuals among communities. We also find that there exists a mobility rate threshold *q_c_*. The epidemic will survive when *q* > *q_c_* and die when *q* < *q_c_*. These results can help understanding the impacts of human travel on the epidemic spreading in complex networks with community structure.

One of the important dynamics on complex networks is epidemic spreading. The community structure in complex networks has considerable influences on the epidemic spreading processes. In this paper, we propose a network model with time-varying community structure. In this model, connections are static within communities and they are dynamic between communities. It is found that the time of the epidemic outbreak depends mainly on the mobility rate of the individuals in this model. We have also derived a critical value with respect to the mobility rate. Our results may be helpful in evaluating the epidemic outbreak time in a community, which contacts with the other infected communities, and controlling the mobility rate of the individuals among communities to prevent the outbreaks of an infection.

## I. INTRODUCTION

Epidemic spreading, including the spreading of human diseases, rumors, and computer viruses, has been one of the most prolific fields in complex network dynamics.^1,2^ Large research effort has recently been devoted to the study of the susceptible-infected-susceptible (SIS) epidemic model, where each vertex belongs to one of two states, either susceptible or infected.^3^ Mathematical analysis on such model has revealed the importance of topology for propagation dynamics, such as lack of an epidemic threshold on scale-free networks.^4,5^ These studies assumed the network evolves more slowly than the diffusion process. The system can be modeled as a static network basing on this assumption, which is effective in many circumstances, such as the spread of the computer viruses, traffic dynamics, and the propagation of some diseases which spread rapidly. However, the propagation of many infectious diseases has comparable time scale as fluctuation of the networks topology, such as tuberculosis, foot and mouth disease among cattle, and various sexually transmitted infections.^6–9^

In recent years, there have been extensive research activities on time-varying networks, or called temporal networks, which evolve on a time scale comparable to the time scale of the propagation process.^10–12^ An additional time dimension is considered in time-varying networks. At every time step, an instance of a time-varying network is formed. All these network instances for many time steps can be aggregated to a static network. An excellent overview of the various applications of temporal networks in many disciplines can be found in Ref. 10. More recently, Hoppe *et al.* have investigated mutual selection in time-varying networks.^11^ Kotnis *et al.* found the existence of an “adaptive threshold,” on adaptive time-varying networks.^12^ Gong *et al.* have investigated the time-varying human mobility patterns with metapopulation epidemic dynamics.^13^ Starnini *et al.* have investigated the immunization strategies for epidemic processes in time-varying contact networks.^14^ All these investigations indicate that time-varying networks play an important role in the investigation of the epidemic spreading that occurs on complex networks.

Real networks, such as the social network, have obvious community structure where the links are dense in a community but sparse between communities. The community structure in complex networks has considerable influences on the epidemic spreading processes. Liu *et al.* found that, compared to the random network, the community network has a broader degree distribution, a smaller threshold of epidemic outbreak.^15^ However, Hébert-Dufresne *et al.* present a model which predicts higher epidemic thresholds for clustered structures than for equivalent random topologies in the case of networks with zero degree correlation.^16^ Wu *et al.* found that the efficiency of epidemic spreading in their model depends mainly on the degree of community. For a fixed degree of community, the efficiency will decrease with increase of the clustering coefficient.^17^ Chen *et al.* proposed a network model with overlapping community structure and investigated the impact of overlapping community structure on SIS epidemic spreading process.^18^ Zhang *et al.* investigated the impact of community structure on a stochastic susceptible-infected-removed (SIR) epidemic.^19^ Considering the difference in the sizes of the infected clusters, inhomogeneity of epidemic spreading has been studied in Refs. 20 and 21.

Despite all these efforts, the impact of time-varying community networks evolution on epidemic spreading has not been well considered. Recently, inspired by data analysis studies about human travel,^22,23^ Skufca *et al.* have introduced a basic model for human mobility that accounts for the different dynamics arising from individuals embarking on short trips and individuals relocating to a new home.^24^ These research efforts provide a new idea for studying time-varying community networks. In this paper, we present a simple networks model with time-varying community structure, and investigate SIS epidemic spreading processes in this model.

The paper is organized as follows. In Sec. II, a network model with time-varying community structure is proposed and the relationship between the time-varying networks and the static networks is given. Then, in Sec. III, we consider the SIS model and implement a numerical simulation to investigate the influences of the mobility rate on dynamic behavior. By applying mean-field approach, the theoretical analysis of the model is performed. Finally, we conclude the paper in Sec. IV.

## II. THE MODEL WITH THE TIME-VARYING COMMUNITY NETWORKS

**FIG. 1.**
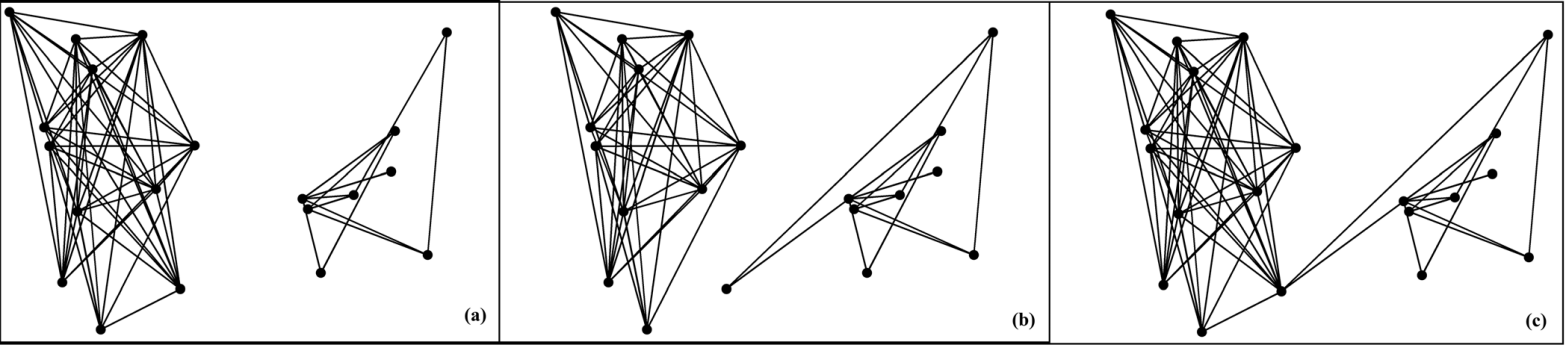
Schematic illustration of the time-varying community network. (a) At first time step, no link exists between two independent communities. (b) At second time step, one individual in the left community jumps into the right community and connects two individuals in the right community. At the same time, this individual breaks all links in the left community. (c) The static community network which aggregates the two forth instances (a) and (b).

Communities in a social network might represent real social groupings, perhaps by background or area. In this paper, we consider the community structure by the regional division. So the same family, school, or the city's populations belong to the same community. Epidemic spreading is rapid in the communities but slow between communities. Due to different communities are apart, it is impossible for individuals to propagate virus to different community at the same time, even if these individuals have connections with many different communities in a static network. So there exists no link among communities at each time step in a time-varying network, but individuals can move among communities, which is similar to human travel in the metapopulation networks.^25,26^ The sum of network instances during all time steps constitutes a static network with community structure. As shown in Fig. 1.

Based on above analysis, the time-varying community network can be constructed as follows:

(1) Consider a total population of *N* individuals who are divided into *m* groups with random *n_i_* (*i* = 1, 2,…, *m*) individuals in each group, and let them satisfy
  $$\sum_{i=1}^{m}n_{i}=N.$$ (1)
(2) At the first time step, in each group *i* we use probability *p_i_* to add a link between every two nodes and let them satisfy
  $$\sum_{i=1}^{m}p_{i}\cdot \frac{1}{2}n_{i}(n_{i}-1)=\frac{N\cdot \left\langle k\right\rangle }{2},$$ (2)
  where ⟨k⟩ is the average degree of the total network.
(3) At the second time step, each individual *j* (*j* = 1, 2,…, *N*) has the probability *q* to jump to other community chosen randomly. During this process, we use probability $p_{i}\times r$ to add a link between the jumped individual and the other individual in the community. At the same time, the jumped individual breaks all links connected with him at the last time step.
(4) Repeat the second time step for *T* times, and then the network returns to the status at the first time step. Repeat this process until the time step set by program. We call *q*, *r*, and *T* as the mobility rate, the connected rate, and the mobility period, respectively.

Now we analyze the static community network, which aggregates all instances formed in the *T* + 1 time steps. If we suppose that the number of the individuals in each group is equal, i.e., $n_{i}=n=N/m$, and $p_{i}$ is constant p, the probability of the connecting links among communities can be written as

$$X=\frac{NqnprT}{n^{2}m(m-1)/2}.$$ (3)

So we get the degree of community defined in Ref. 12

$$\sigma =\frac{p}{X}=\frac{m-1}{2qrT}.$$ (4)

Obviously, for a certain number of the communities m, σ, and qrT have an inverse relationship. Here, we discuss the epidemic spreading on this model.

## III. THE SIS EPIDEMIC SPREADING

We mainly analyze the impact of the mobility rate q on the epidemic spreading using SIS model. In the SIS model, individuals have two possible states: susceptible and infected. Assume that each susceptible neighbor of an infected individual has a probability λ to be infected. If a susceptible individual has $k_{\inf}$ infected neighbors, then at each time step this susceptible individual will become infected with probability $1-{\left(1-\lambda \right)}^{k_{\inf}}$. At the same time, each infected individual will become susceptible at rate μ at each time step. The basic notion in epidemiology is an epidemic threshold $\lambda_{c}$. The epidemic spreads and becomes endemic for $\lambda >\lambda_{c}$ and dies for $\lambda <\lambda_{c}$. From the theory of probability,^27^ we have $\lambda_{c}=\mu /\left\langle k\right\rangle$.

In the time-varying community networks model, at each time step, none of link exists among different communities. If the mobility rate q=0, for a specific community i, its epidemic sub-threshold

$$\lambda_{c}^{i}=\frac{\mu }{\left\langle k_{i}\right\rangle }=\frac{\mu }{p_{i}(n_{i}-1)}.$$ (5)

The epidemic in a specific community *i* will survive when $\lambda >\lambda_{c}^{i}$ and die when $\lambda <\lambda_{c}^{i}$. Supposing there is only one seed in the beginning, the epidemic spreading will be confined within the community where the seed is chosen.

However, when q>0, the situation will be totally changed because individual may jump to other communities. First, when $\lambda >\lambda_{c}^{i}(i=1,2,...,m)$, even having only one seed in the beginning, the epidemic can spread into all communities. We find that the time of the epidemic outbreak in the communities where there is no seed is intensively dependent on the mobility rate *q*. Second, when $\lambda <\lambda_{c}^{i}(i=1,2,...,n;n<m)$, a mobility rate threshold $q_{c}$ is considered. The epidemic in the community *i* where $\lambda <\lambda_{c}^{i}$ can survive when $q>q_{c}$ because of the jump and die when $q<q_{c}$. Third, when $\lambda <\lambda_{c}^{i}(i=1,2,...,m)$, the epidemic will die in all communities. Here, we analyze epidemic spreading in the complex networks with time-varying community structure in the first two cases. To be brief, let us set *m* = 2, *T* = 1, and *r* = 0.1.

### A. $\lambda \;>\;\lambda_{c}^{i}(i=1,2)$

**FIG. 2.**
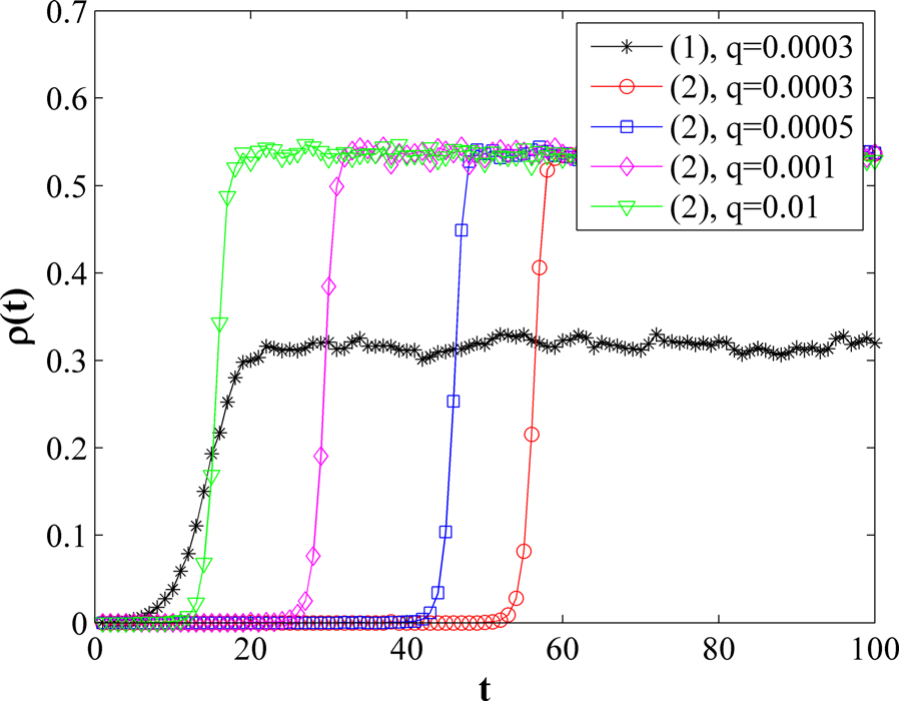
Density of infected nodes ρ as a function of *t* in two communities with different mobility rates *q*. In the legend, the symbol (1) denotes the first community and the symbol (2) denotes the second community.

Without lack of generality, we take N=2000, $n_{1}=800$, $n_{2}=1200$, ⟨k⟩=40, $p_{1}=0.0206$, and $p_{2}=0.0464$. Obviously, these parameters satisfy Eqs. (1) and (2). Let us set μ=0.1. We can calculate $\lambda_{c}^{1}=0.0061$ and $\lambda_{c}^{2}=0.0018$ from Eq. (5). We take $\lambda =0.04>\lambda_{c}^{i}(i=1,2)$. In the simulation, initially, we randomly chose one individual in the first community to be infected while all the rest in two communities were susceptible. As shown in Fig. 2, the curve with black asterisks represents the density of infected nodes in the first community as a function of time with mobility rate *q* = 0.0003 and the others represent evolution of infected nodes in the second community with different mobility rate from 0.0003 to 0.01. Obviously, the epidemic first outbreaks in the first community and then propagate into the second community. The time of epidemic outbreak in the second community decrease with the increase of the mobility rate *q*. We do not plot the curve for the other mobility rate in the first community because they overlap almost completely with the curve for *q* = 0.0003. The values applied above in the model are chosen mainly by experiment and through experience. They are representative for illuminating our results. Similar results can be obtained when other suitable values are chosen. A deeper understanding of the detailed time evolution of epidemic transmission is a prerequisite to finding optimal strategies to prevent outbreaks of an infection. Consequently, we analyze it in detail.

Due to $\lambda >\lambda_{c}^{2}$, the epidemic can outbreak in the second community only if there is one individual, which is infected by the individuals which move from the first community to second community. At each time step, the number of the infected individuals which move from the first community to second community are $n_{1}q\rho_{1}(t)$, where $\rho_{1}(t)$ represents the density of the infected individuals in the first community at the time step *t* and *q* is mobility rate among communities and $n_{1}$ is the total individual number in the first community. According to mean field theory, $\rho_{1}(t)$ satisfy equation ${\dot{\rho }}_{1}(t)=-\mu \rho_{1}(t)+\lambda \left\langle k_{1}\right\rangle \rho_{1}(t)(1-\rho_{1}(t))$, which has a simple solution

$$\rho_{1}(t)=\frac{a/b}{1+ce^{-at}},$$ (6)

where $a=\lambda \left\langle k_{1}\right\rangle -\mu$, $b=\lambda \left\langle k_{1}\right\rangle$ and

$$c=\frac{a-\rho_{1}(0)b}{\rho_{1}(0)b}.$$ (7)

$\rho_{1}(0)$ represents the density of infected individuals at t=0, and in this example $\rho_{1}(0)=1/n_{1}$. In the $n_{1}q\rho_{1}(t)$ individuals which move from the first community to the second community, each individual connect $n_{2}p_{2}r$ individuals in the second community. Each connected individual in the second community has probability λ to be infected. Supposing the probability of one individual in the second community infected in $t_{c}$ time step is 100%, we can write

$$\int_{0}^{t_{c}}n_{1}q\rho_{1}(t)n_{2}p_{2}r\lambda dt=1.$$ (8)

Solving this equation, we can get

$$t_{c}=\frac{\ln(e^{\ln(1+c)+b/(\lambda wq)}-c)}{a},$$ (9)

where $w=n_{1}n_{2}p_{2}r$. Finally, we can get the outbreak time of the epidemic in the second community

$$T_{c}=2(t_{c}+t_{0})=2(\frac{\ln(e^{\ln(1+c)+b/(\lambda wq)}-c)}{a}+\frac{\ln\;c}{a}),$$ (10)

where $t_{0}=\ln c/a$ is the time when the number of infected individuals in the second community increases from one to half of stabilized value. Due to the mobility period T=1, the migration individuals must come back to the initial place after they move to another community. So the actual time of the epidemic outbreak in the second community is $2(t_{c}+t_{0})$.

**FIG. 3.**
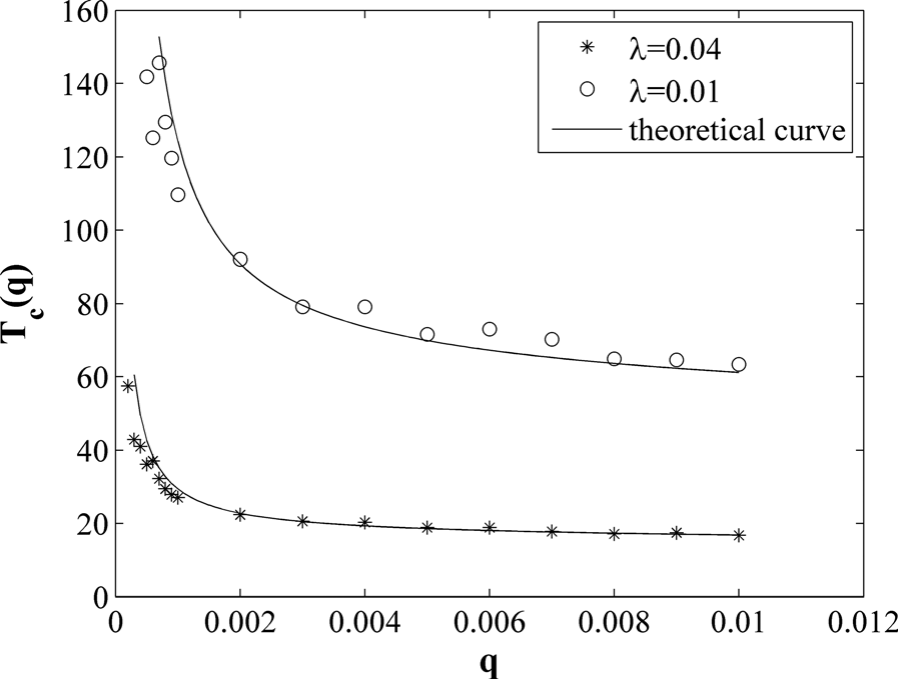
The epidemic outbreak time *T*_c_ versus the mobility rate *q*, where the symbols represent the results of numerical simulations and the lines represent the results from Eq. (10). The results of numerical simulations are averaged on 100 realizations.

For checking the above analysis, let us make numerical experiments. We determine $T_{c}$ by checking the number of infected individuals of the second community, which reach half of stabilized value at the time step $T_{c}$. We structure the time-varying network with the same parameters as used in Fig. 2, but we set λ two different values 0.01 and 0.04, and we change the mobility rate q from 0.0003 to 0.01. We take average on a number of realizations. As shown in Fig. 3, the circles and asterisks denote the case of λ=0.01 and λ=0.04, respectively. The two lines represent results calculated from Eq. (10) with two different λ values. Obviously, the numerical simulations are consistent with the theoretical prediction.

### B. $\lambda_{c}^{2}\;<\;\lambda \;<\;\lambda_{c}^{1}$

**FIG. 4.**
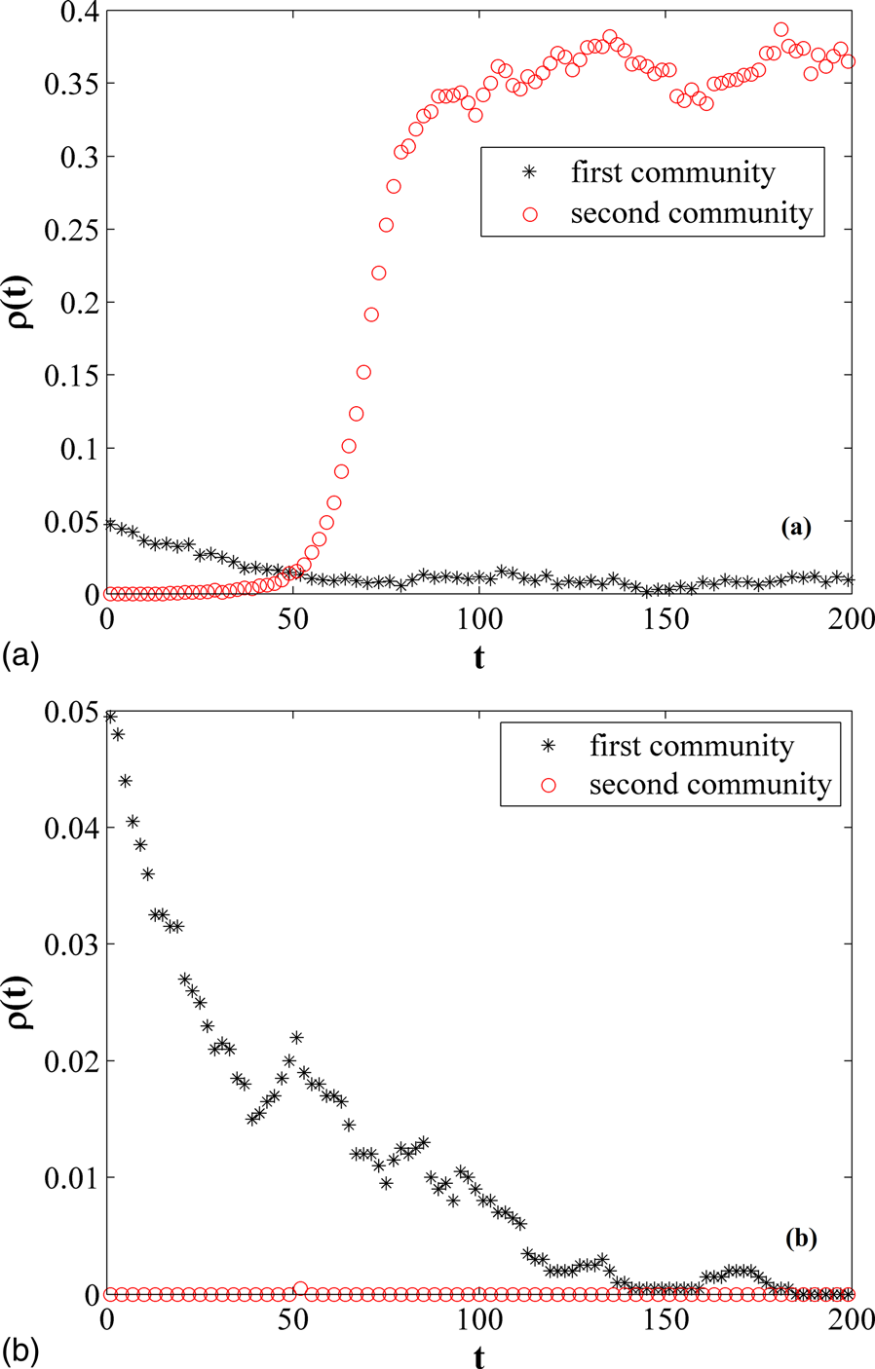
Density of infected nodes ρ as a function of *t* in two communities with λ = 0.005. The initial number of infected seeds *x*(0) = 100, which are randomly chose in the first community. (a) *q* = 0.008 and (b) *q* = 0.001.

In this case, the epidemic will die in the first community when the mobility rate is too low. However, if the mobility rate is enough high, the epidemic can spread into the second community before it die, and the epidemic can also survive in the first community because of the infected individuals jumping from the second community to the first community. For checking this idea, let us perform experiments. We structure the time-varying networks with the same parameters as used in Fig. 2, but λ=0.005, which is larger than $\lambda_{c}^{2}=0.0018$ and is smaller than $\lambda_{c}^{1}=0.0061$. And we set the initial number of infected individuals x(0)=100, which is randomly chosen in the first community. So we can calculate $\rho_{1}(0)=x(0)/n_{1}=0.125$, $\rho_{2}(0)=0$, and ρ(0)=x(0)/N=0.05, where $\rho_{1}(0)$, $\rho_{2}(0)$, and ρ(0) represent the density of infected individual in the first community, second community, and total network, respectively. Fig. 4 shows the evolution process of ρ(t) in two communities. The black asterisks represent the density of infected individuals in the first community as a function of time and the red circles represent evolution of infected individuals in the second community. Obviously, as shown in Fig. 4(a), the epidemic outbreaks at *t* = 60 approximately for *q* = 0.008 in the second community and also survives in the first community, although the number of infected individuals is quite low in the first community. However, for *q* = 0.001, the number of infected individuals is reduced gradually to zero in the first community and the epidemic do not outbreak all along in the second community, which can be seen from Fig. 4(b).

Now let us analyze theoretically how the mobility rate influences epidemic spreading in this case. Duo to $\lambda <\lambda_{c}^{1}$, there exists a time step $t_{1}$, the epidemic will die when $t=t_{1}$ in the first community. The epidemic can survive only if the infected individuals can move into the second community and at least one susceptible individual in the second community is infected before $t=t_{1}$. First, we calculate $t_{1}$. According to Eq. (6), $\rho_{1}(t)$ is reduced gradually to be close to zero when *a* < 0. So we can set $\rho_{1}(t_{1})$ a very small number. Here, we set $\rho_{1}(t_{1})=0.0001$ and solving Eq. (6) we get

$$t_{1}=\frac{\ln(\frac{10000a-b}{bc})}{-a}.$$ (11)

From Eqs. (9) and (11), and considering $t_{1}=2t$ when $q=q_{c}$, we can get

$$q_{c}=\frac{\left\langle k_{1}\right\rangle }{n_{1}n_{2}p_{2}r(\ln(\sqrt{\frac{\lambda \left\langle k_{1}\right\rangle c}{1000(\lambda \left\langle k_{1}\right\rangle -\mu )-\lambda \left\langle k_{1}\right\rangle }}+c)-\ln(1+c))},$$ (12)

where *c* is defined as same as Eq. (7).

**FIG. 5.**
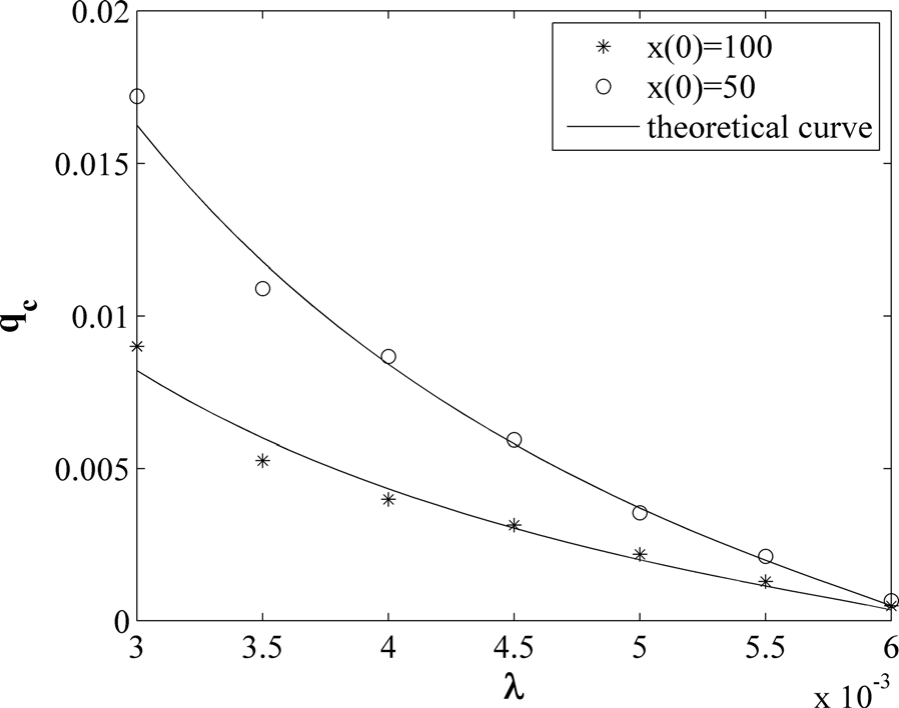
The mobility rate threshold *q*_c_ versus the infection rate *λ* with the different initial number of infected individuals where the symbols represent the results of numerical simulations and the lines represent the results from Eq. (12). The results of numerical simulations are averaged on 100 realizations.

In numerical experiments, we set *x*(0) = 50 and 100, and set *λ* = 0.03 to 0.06, and then increase gradually mobility rate *q* from zero. When the mobility rate *q* increases to the mobility rate threshold *q*_c_, the epidemic outbreaks in the second community. For each set of *x*(0) and *λ*, we take average on 100 realizations. As shown in Fig. 5, the circles and asterisks represent the experiment results for *x*(0) = 50 and 100, respectively. The lines represent the results from Eq. (12). It shows that for a fix *λ*, the mobility rate threshold *q*_c_ is approximately inversely proportional to the initial number of infected individuals in the first community *x*(0). However, for a fix *x*(0), *q*_c_ decrease intensively with the increase of the infected rate *λ*. For example, for *x*(0) = 100, *q*_c_ decrease from 0.09 to 0.000482 when *λ* increase from 0.003 to 0.006. Our numerical simulations have confirmed the prediction by Eq. (12). These results may be applied to the real situation. When an infection occurs in a community, we usually use the segregate method, which is to break the connection between the infected community and the other ones. But usually, human travel is unavoidable. In this case, we can evaluate a suitable mobility rate to protect the non-infected communities.

## IV. CONCLUSIONS

We propose a network model with time-varying community structure. In this model, connections are static within communities and they are dynamic between communities. The impact of the mobility rate on the epidemic spreading is studied. It is found that the epidemic can outbreak in the community in which there exists no infected individual initially and the outbreak time decreases with the increasing of the mobility rate. More importantly, we have also derived a critical value with respect to the mobility rate. The epidemic outbreaks with the mobility rate larger than the critical value and dies with the mobility rate smaller than the critical value in all communities. Our results may be helpful in evaluating the epidemic outbreak time in a community which contacts with the other infected communities, and controlling the mobility rate of the individuals among communities to prevent the outbreaks of an infection.
